# Supplementary material for: Acetone Sensors Based on Al-Coated and Ni-Doped Copper Oxide Nanocrystalline Thin Films
Source: Sensors (Basel). 2024 Oct 11;24(20):6550. doi: 10.3390/s24206550 (PMC11511153; doi:10.3390/s24206550)
Supplement: Supplementary file 1 [file sensors-24-06550-s001.zip › sensors-3183047-Supplementary Materials.pdf]

Supplementary Materials

Acetone Sensors Based on Al-Coated and Ni-Doped Copper Oxide Nanocrystalline Thin Films

Dinu Litra [1,2], Maxim Chiriac [1,2], Nicolai Ababii [1,2] and Oleg Lupan [1,2,3,4,*]

1 Center for Nanotechnology and Nanosensors, Technical University of Moldova, 168 Stefan cel Mare Av., MD-2004 Chisinau, Moldova; dinu.litra@mib.utm.md (D.L.); maxim.chiriac1@mib.utm.md (M.C.); nicolai.ababii@mib.utm.md (N.A.)
2 Department of Microelectronics and Biomedical Engineering, Technical University of Moldova, 168 Stefan cel Mare Av., MD-2004 Chisinau, Moldova
3 Department of Physics, University of Central Florida, Orlando, FL 32816-2385, USA
4 Chair for Advanced Materials, Department for Materials Science, Kiel University, Kaiserstr. 2, D-24143 Kiel, Germany
* Correspondence: oleg.lupan@mib.utm.md

Supplementary Materials

Appendix A

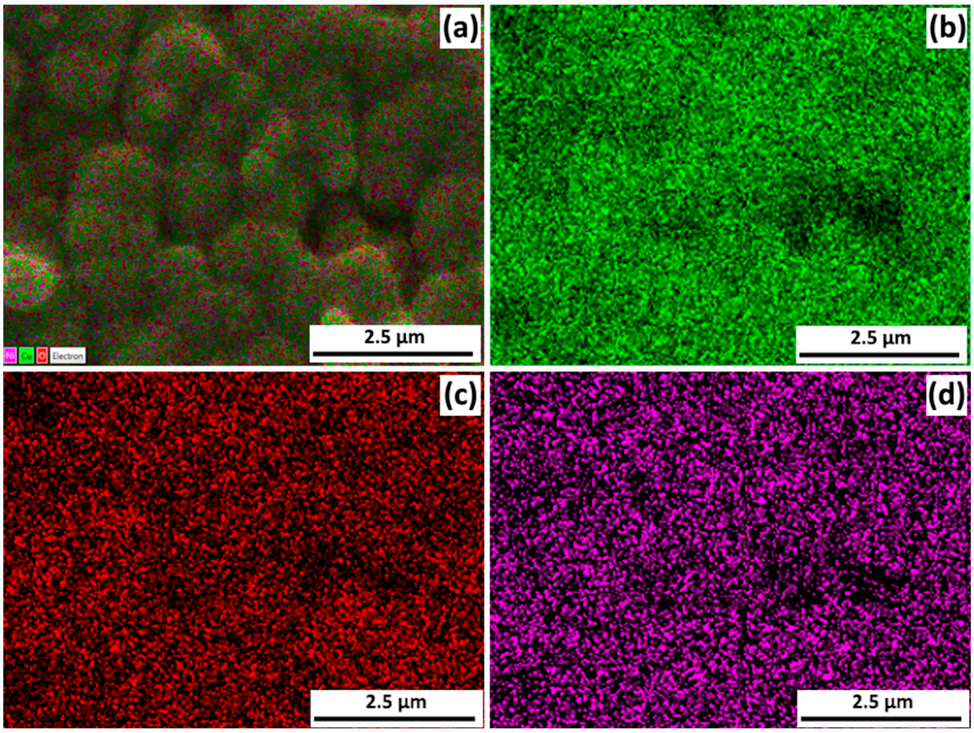

Figure S1. Layered and composition images obtained through EDX mapping of CuO:Ni nanostructures (b) Cu Lα1_2; (c) O Kα1; (d) Ni Lα1_2

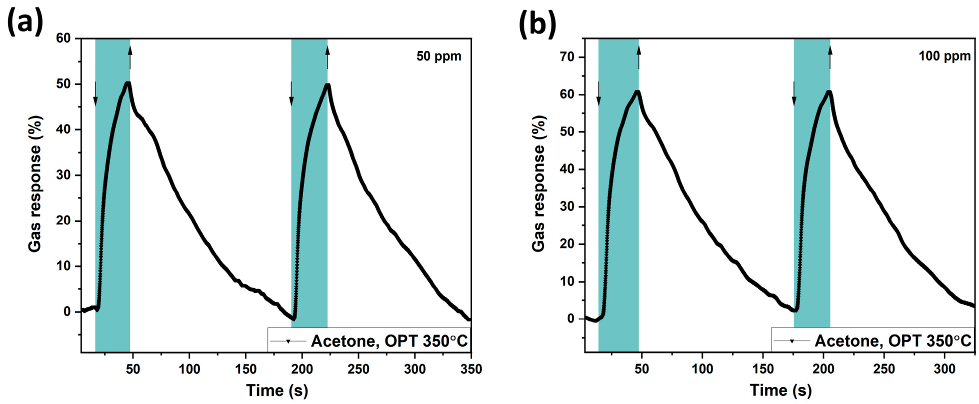

Figure S2. The dynamic response to acetone of Al/CuO:Ni structures at 350 °C at pulses concentrations of 50 ppm (a) and 100 ppm (b) after 242 days.
